# Multiple regions of sensorimotor cortex encode bite force and gape

**DOI:** 10.3389/fnsys.2023.1213279

**Published:** 2023-09-22

**Authors:** Fritzie I. Arce-McShane, Barry J. Sessle, Yasheshvini Ram, Callum F. Ross, Nicholas G. Hatsopoulos

**Affiliations:** ^1^Department of Oral Health Sciences, School of Dentistry, University of Washington, Seattle, WA, United States; ^2^Graduate Program in Neuroscience, University of Washington, Seattle, WA, United States; ^3^Faculty of Dentistry and Department of Physiology, Faculty of Medicine, University of Toronto, Toronto, ON, Canada; ^4^Department of Organismal Biology and Anatomy, University of Chicago, Chicago, IL, United States

**Keywords:** sensorimotor cortex, force, gape, encoding, decomposition, population activity, orofacial

## Abstract

The precise control of bite force and gape is vital for safe and effective breakdown and manipulation of food inside the oral cavity during feeding. Yet, the role of the orofacial sensorimotor cortex (OSMcx) in the control of bite force and gape is still largely unknown. The aim of this study was to elucidate how individual neurons and populations of neurons in multiple regions of OSMcx differentially encode bite force and static gape when subjects *(Macaca mulatta)* generated different levels of bite force at varying gapes. We examined neuronal activity recorded simultaneously from three microelectrode arrays implanted chronically in the primary motor (MIo), primary somatosensory (SIo), and cortical masticatory (CMA) areas of OSMcx. We used generalized linear models to evaluate encoding properties of individual neurons and utilized dimensionality reduction techniques to decompose population activity into components related to specific task parameters. Individual neurons encoded bite force more strongly than gape in all three OSMCx areas although bite force was a better predictor of spiking activity in MIo vs. SIo. Population activity differentiated between levels of bite force and gape while preserving task-independent temporal modulation across the behavioral trial. While activation patterns of neuronal populations were comparable across OSMCx areas, the total variance explained by task parameters was context-dependent and differed across areas. These findings suggest that the cortical control of static gape during biting may rely on computations at the population level whereas the strong encoding of bite force at the individual neuron level allows for the precise and rapid control of bite force.

## Introduction

Primate feeding relies on the coordination of tongue and jaw movements and the precise control of the generation of tongue and bite forces at varying distances of jaw depression (i.e., gape) during biting, chewing, and swallowing. Bite force control is important for intra-oral breakdown of food into a bolus that is safe to swallow and easy to digest while minimizing the probability of tooth breakage and excessive tooth wear. Likewise, gape has to be controlled to accommodate ingestion and manipulation of food by the lips, tongue, and teeth during ingestion, chewing, bolus transport and swallowing. Indeed, the wide range of disorders and dysfunctions affecting the feeding system pose significant challenges for human health and enjoyment of life, including tooth loss, masticatory dysfunctions, dysphagia, and pain states such as temporomandibular disorders and trigeminal neuralgia (Hamdy et al., 1998; Sessle et al., 2005; Khedr and Abo-Elfetoh, 2010; Nguyen et al., 2011; Murdoch et al., 2012; Trulsson et al., 2012). A part of the cerebral cortex termed the orofacial sensorimotor cortex (OSMcx) is crucial for controlling orofacial sensorimotor functions, yet despite the importance of feeding behavior for human health and well-being, little is known about the role of the OSMcx in the control of bite force and gape. This limited knowledge hampers our ability to leverage the full potential of OSMcx for the development of therapies and treatments and also constrains our understanding of the role of OSMcx in feeding system evolution.

The OSMcx, which includes the primary motor (MIo), primary somatosensory (SIo), and cortical masticatory (CMA) areas, plays a crucial role in the control of complex oral sensorimotor behaviors so as to effect functionally critical, coordinated movements such as those associated with feeding and speech (Sessle et al., 2005; Sessle, 2011; Avivi-Arber and Sessle, 2018). Several decades of research using intracortical microstimulation (ICMS), receptive field (RF) mapping, multi-electrode array recordings, and ablative procedures suggest that these three areas play important roles in these behaviors. For example, ICMS in MIo, SIo, or CMA can evoke relatively simple movements of orofacial muscles (e.g., jaw opening, tongue protrusion) as well as more complex movements such as chewing and swallowing (Huang et al., 1988, 1989b; Martin et al., 1997, 1999; Hatanaka et al., 2005; Laurence-Chasen et al., 2018). Neurons in MIo and SIo have been shown to modulate their activity during feeding and performance of orofacial tasks such as the generation of tongue-protrusive force or bite force, to encode the direction and magnitude of tongue-protrusive force, to form coherent networks within and across these areas in a reciprocal manner, and to undergo learning-induced plasticity (Murray and Sessle, 1992a,b; Lin et al., 1994; Arce et al., 2013; Arce-McShane et al., 2016, 2019; Liu et al., 2019). Many of these neurons have orofacial mechanosensitive RFs and the sensory inputs from their RFs are used to modulate bite and tongue forces (Huang et al., 1988, 1989b; Lin and Sessle, 1994; Toda and Taoka, 2002, 2004; Cerkevich et al., 2014). In addition, a role for OSMcx in orofacial motor control is indicated by studies showing that reversible cold-block or ablation of OSMcx disrupts various elements of feeding performance (Luschei and Goodwin, 1975; Murray et al., 1991; Lin et al., 1993, 1998; Narita et al., 2002; Yamamura et al., 2002; Yao et al., 2002).

While these findings in animals indicate an important role for OSMcx in the control of feeding and related orofacial motor behaviors, it is unknown how functionally diverse neuronal populations in three different cortical areas (i.e., MIo, SIo, and CMA) might encode gape and bite force simultaneously because the activity from these areas has not been recorded simultaneously when both bite force and gape parameters are controlled. Here, we present novel data on the role of OSMcx of macaque monkeys in the control of these two critically important behavioral variables in mammalian feeding. The aim of this study was to elucidate how individual neurons and population of neurons in multiple regions of OSMcx differentially encode bite force and gape when subjects *(Macaca mulatta)* generated different levels of bite force at varying static gapes during a biting task.

## Materials and methods

For reference, Table 1 provides a list of acronyms used in this paper.

**Table 1:** List of acronyms used.

**AUROC**	**Area under the receiver operating characteristic curve**
CMA	Cortical masticatory area
dPCA	Demixed principal components analysis
FO	Force onset
GLM	Generalized linear model
ICMS	Intracortical microstimulation
MIo	Primary motor
OSMcx	Orofacial sensorimotor cortex
PETH	Peri-event time histogram
RF	Receptive field
SE	Standard error
SIo	Primary somatosensory

### Subjects

Data were collected from two adult female rhesus macaques (*Macaca mulatta*), H (7.5 kg) and M (5.8 kg). While the craniofacial features of females tend to be smaller than males, there is no evidence that we know of that would indicate a neurophysiological difference in orofacial function between males and females. Likewise, menstrual conditions were not controlled due to unknown effects of menstruation on activity of neurons in the orofacial sensorimotor cortex. All protocols were approved by the University of Chicago Animal Care and Use Committee and complied with the National Institutes of Health *Guide for the Care and Use of Laboratory Animals*.

### Behavioral task

**Figure 1:**
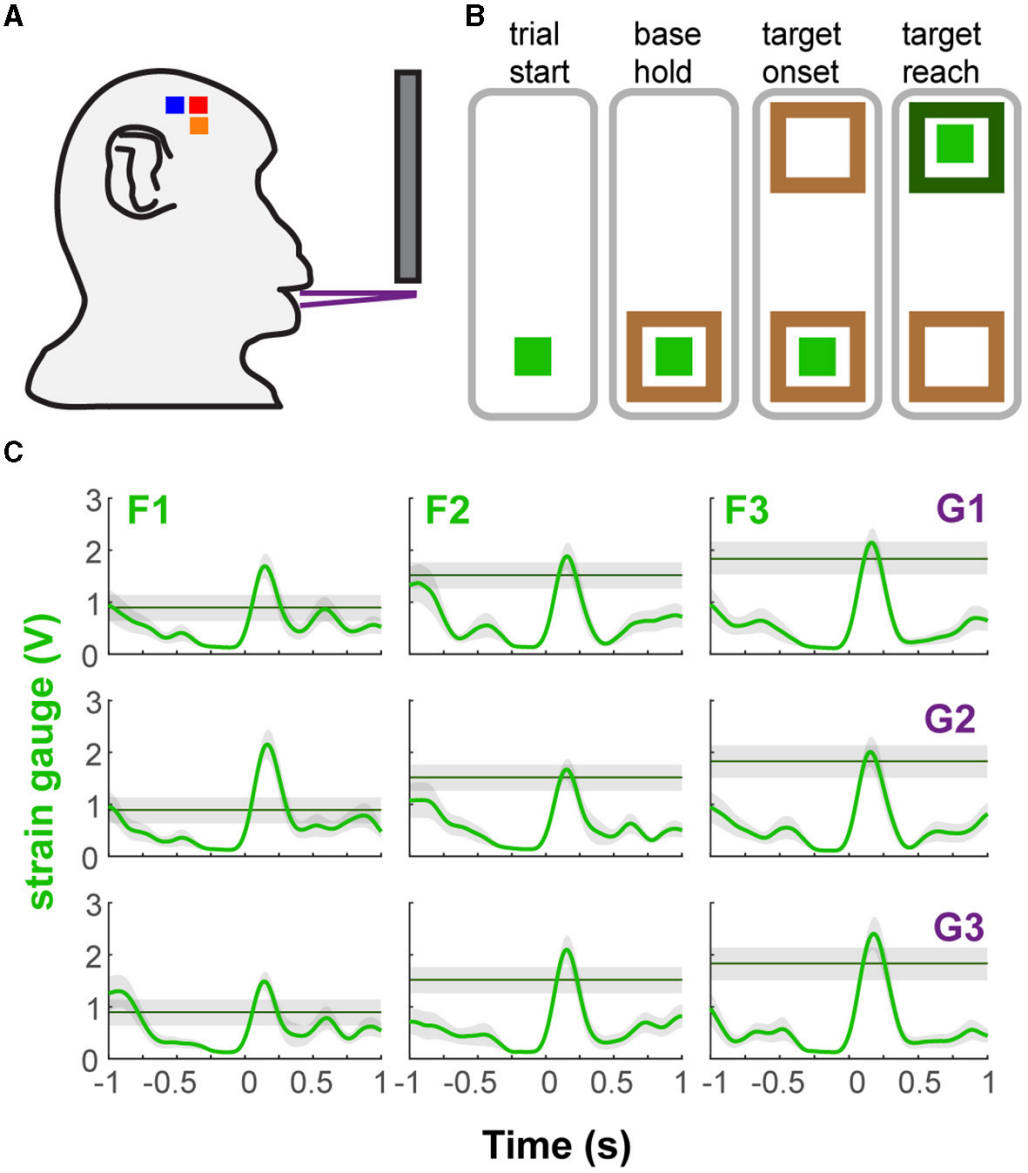
Behavioral task and performance. **(A)** Diagram of the bite force task apparatus. **(B)** Sequence of events in a trial of the bite force task. The light green square represents the force cursor while the brown and green boxes represent the base and force targets. The animals were presented with one of three target positions at one of three gape distances in each trial. **(C)** Example bite force profiles of each trial type based on gape and required bite force level (shaded area covers ± 0.25 V from target line). There was a minimal hold time at the target (0.1 s), allowing the subject to go past the required force level. Shown for subject H as mean bite force and ± 1 SE (shaded area), across all trials of a trial type, during ±1 s relative to force onset (FO). G1, G2, G3 correspond to increasing gape distances whereas F1, F2, F3 correspond to increasing levels of required bite force.

The two naïve monkeys were trained to perform a behavioral task that approximates a natural incisor biting behavior requiring the generation of different levels of bite force at varying static gapes (i.e., jaw depression distances) (Figure 1A). The bite force plates were computer-controlled to open at one of three gapes prior to the start of a behavioral trial (Figure 1B). The bite plates remained in that configuration for the entire length of the trial. Strain gauges bonded to the bite plates recorded the bite force produced by the teeth engaging the bite force plate. Nine combinations of required bite force (3 levels) and gape (3 distances) composed the nine different trial types (Figure 1C and Supplementary Figure S1A). The presentation order of gapes was randomized in subject M and blocked in subject H. In blocked presentation, three force levels were randomly paired with a single gape before moving on to another gape. Subjects were rewarded with juice after holding the force level at the target. A detailed description of the task flow can be found in the Supplementary material.

### Electrophysiology

Under general anesthesia, each subject was chronically implanted with silicon-based arrays of 64 or 100 microelectrodes (BlackRock Microsystems, Salt Lake City, UT) in MIo, SIo and CMA of the left hemisphere (Supplementary Figure S1B). The microelectrodes on the array were separated from their immediate neighbors by 400 μm and their length was 1.5 mm for arrays implanted in MIo and 1.0 mm for SIo and CMA. Implantation sites were verified based on surface landmarks and exhibited movements of the tongue or fingers evoked by monopolar surface stimulation of MIo (50 Hz, 200 μs pulse duration, 2–5 mA) during the surgical procedure. Signals from both arrays were amplified with a gain of 5,000, simultaneously recorded digitally (16-bit) with a sampling rate of 30 kHz and hardware-filtered using a high-pass filter fixed at 1 Hz first, followed by a low-pass filter with 7.5 kHz cut-off (Grapevine, Ripple LLC, Salt Lake City, UT). Spike data streams were digitally filtered with a high-pass filter at 250 Hz. Spike waveforms were stored and sorted offline using Offline Sorter (Plexon, Dallas, TX). Data from array channels with no signal or with large amounts of 60 Hz line noise were excluded.

### Data analysis

Two datasets, one from each subject, were used in the main analyses (H: 20160209; M: 20161020). Spiking activity of all individual neurons recorded simultaneously from MIo, SIo, and CMA in one session was used in all neural analysis (Table 2).

**Table 2:** Number of neurons included in GLM and dPCA analyses.

	**Subject M**	**Subject H**
MIo	70	123
SIo	62	64
CMA	41	78

#### Generalized linear model

To determine the relative importance of bite force and static gape in predicting the firing of neurons and to compare encoding properties among OSMCx areas, we used GLM to predict the time-varying spiking activity of each neuron. The GLM approach allows us to measure how well a model predicts the probability that a neuron fires a spike in a small sampling window (4 ms) based on different combinations of extrinsic covariates (i.e., bite force and gape) and intrinsic covariates (i.e., spike history). The time window used in the analysis was ±500 ms relative to force onset (FO).

##### Extrinsic covariates

For gape, we used the gape distances which were adapted to the subject's mandibular length (H: 11, 14, 17 mm; M: 8, 11, 14 mm). For bite force, we included bite force magnitude at eight different time lags from −156 ms (i.e., force leads spikes by 156 ms) to 208 ms (i.e., force lags spikes by 208 ms) in 52 ms steps relative to the spike sampling window. We used multiple time lags because multi-lag GLM models using kinematic features have been shown to provide higher predictive power than models that include only a single, optimal lag (Hatsopoulos et al., 2007; Saleh et al., 2010, 2012; Takahashi et al., 2017). We also included an interaction term for gape and bite force to evaluate whether encoding of the interaction between these two features was better than encoding of each feature separately. Thus, we used a total of 17 input features (1 gape, 8 forces, 8 interactions between gape and force) as extrinsic covariates.

##### Spike history

The current spiking activity of a neuron might also be affected by its own spiking activity in the past due to intrinsic physiological properties such as absolute and relative refractory periods. Thus, we included the neuron's spike history as an intrinsic covariate. To account for short (16 ms), medium (44 ms), and long (108 ms) time scale effects of the neuron's own spike history, we filtered binary spike trains with raised cosine basis functions (Supplementary Figure S2). A logit link function was used to relate the logarithm of the firing intensity (which is approximately equivalent to the spiking probability given the small 4 ms spike-sampling window) to a linear combination of covariates, expressed as:


$$\begin{array}{l} \log\left[\frac{p_{n}\left(t\right)}{1-p_{n}\left(t\right)}\right]\;=\;\beta_{0}+\sum_{j\;=\;1}^{J}\beta_{j}^{H}H_{j}(t)+\sum \beta^{G}G\left(t\right)\\ \;+\sum_{k\;=\;1}^{K}\beta_{k}^{F}F_{k}(t-\tau_{k})+\sum_{k\;=\;1}^{K}\beta_{k}^{GF}GF_{k}(t-\tau_{k}) \end{array}$$

where *p*_*n*_(*t*) is the probability that neuron *n* fires a spike at time *t*, β_0_ represents the baseline probability that the neuron will spike, *H*_*j*_(*t*) is the value of the *j*^th^ (of *J*) spike history timescale at time *t, G* is the gape distance at time *t*, *F*_*k*_(*t*−τ_*k*_) is the bite force at time *t*−τ_*k*_, where τ_*k*_ is the *k*^*th*^ (of *K*) lead or lag time against the spike time at *t*, and *GF*_*k*_(*t*−τ_*k*_) is the interaction covariate at time *t*−τ_*k*_, and each covariate's weight $\beta_{j}^{H}$, *β*^*G*^, $\beta_{k}^{F}\;$and $\beta_{k}^{GF}$, respectively.

##### Assessing the relative importance of each covariate

We used different models based on the combination of input features used to predict a neuron's firing. The full model includes all input features (bite force, gape, interaction, and the spike history of the neuron) while the reduced models have up to three of the input features removed (i.e., gape removed, bite force removed, and both force and gape removed, only force, only gape, only interaction, only spike history). To measure the goodness of fit of the encoding model, we compared the area under the receiver operating characteristic curve (AUROC) for ten folds of cross-validated test data (i.e., 10 distinct sets of test trials that were not used to build the model) against chance level (Hatsopoulos et al., 2007; Truccolo et al., 2009; Saleh et al., 2010, 2012; Takahashi et al., 2017).

#### Demixed principal components analysis

To investigate how activity of neuronal populations in MIo, SIo, and CMA might distinguish between behavioral parameters, we used dPCA (Kobak et al., 2016) to decompose the dependencies of the population activity, ***X***, into components of time-dependent and task-dependent parameters: the task-independent parameter of *time*, *X*_*T*_ (for activity related to the progression through the behavioral trial), the task-dependent parameters of *bite force*, *X*_*F*_, and *gape*, *X*_*G*_, and the *interaction* between them, *X*_*I*_:


$$\begin{array}{l} \text{X}\;=\;X_{T}+\;X_{G}+\;X_{F}+\;X_{I\;}+\;X_{noise}\; \end{array}$$

where ***X*** is the full data matrix with N rows of neurons which contain smoothed spike train of the *n*^th^ neuron for all task conditions and all trials. *X*_*T*_, *X*_*G*_, *X*_*F*_, *X*_*I*_ are the linear decompositions (i.e., components) of X into parameter-specific averages. dPCA then finds separate decoder (*D*) and encoder (*F*) matrices for each of these terms, φ , by minimizing the loss function: ${\;L}_{dPCA}\;=\;\underset{\varphi }{\sum }||X_{\varphi }-F_{\varphi }\;D_{\varphi }X||{}^{2}$. To assess whether the condition tuning of individual dPCA components was statistically significant, we implemented the decoding method provided in the dPCA Toolbox (Kobak et al., 2016) where classification accuracy was measured for each time point of a behavioral trial by using the decoding axes of the first components of each marginalization, i.e., the first component of bite force was used to classify force levels, the first component of gape to classify gapes, and the first interaction component to classify all nine trial types. The dPCA Toolbox uses cross-validation to measure time-dependent classification accuracy and a shuffling procedure to assess classification accuracy that is significantly above chance. We used 1,000 iterations of stratified Monte Carlo leave-group-out cross-validation wherein on each iteration, one trial for each neuron in each condition was held out to form the test set and the remaining trials to form a training set. We used 500 iterations for the shuffling procedure. Default number of iterations for cross-validation and shuffles used in Kobak et al. (2016) was 100. To verify that the number of iterations did not affect the acceptance limits significantly, we also performed 100 cross-validation with 10,000 shuffling iterations for the CMA dataset of monkey H which took about 12 h to run on a 2.9 GHz on a quad-core. The chance level was slightly higher in the 10,000 shuffles compared to the chance level obtained from 500 shuffles. In both cases, classification accuracies were not above chance levels. Based on this result, we think that using 1,000 cross-validation and 500 shuffling iterations was sufficient to estimate chance level. The mean classification accuracy (from 1000 repetitions of cross-validation) per time bin (50 ms) was deemed significant when the corresponding actual mean classification accuracy exceeded all 500 shuffled decoding accuracies. This would, therefore, be equivalent to a *p*-value of 0.002.

We used the nonparametric Kruskal-Wallis one-way analysis of variance and the Bonferroni test for *post-hoc* multiple paired comparison with significance level set at *p* < 0.05, unless otherwise noted. For reporting *p*-values, we used either the equality sign for exact values (e.g., *p* = 0.01) and the inequality sign when the *p*-value was rounded to the next whole number or when reporting the highest value for a group of tests for brevity (e.g., *p* < 5x10^−8^)^.^ All other analyses were performed using built-in and user-defined functions in Matlab (Mathworks, Inc.).

## Results

**Figure 2:**
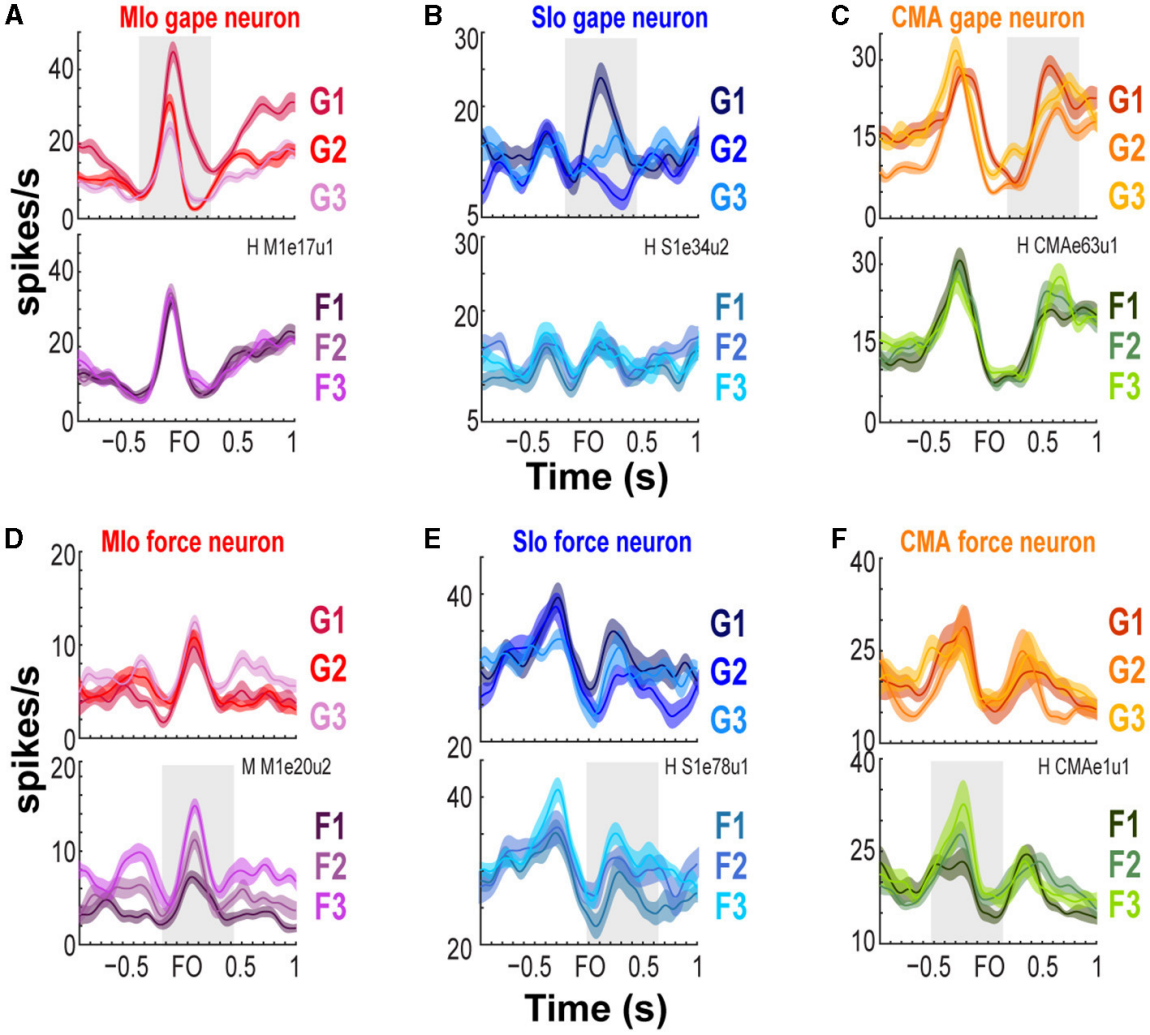
Modulation of spiking activity of single units in MIo, SIo, and CMA during task performance. **(A–C)** Peri-event time histograms (PETHs and ±1 SEM smoothed by a 50-ms Gaussian kernel) of individual gape-related neurons simultaneously recorded in MIo, SIo, and CMA, respectively. To illustrate whether a neuron is gape-related or force-related, trials used to plot the PETHs were grouped according to gape (top row, pooling over force levels) or required bite force levels (bottom row, pooling over gape distances). For example, in **(A)** MIo gape neuron shows modulation of peak activity with different gape distances (top row) but not with different levels of force (bottom row). **(D–F)** As in **(A–C)**, but for neurons whose spiking activity varied more with the varying degrees of bite force than gape.

We trained two naïve monkeys to perform an incisor biting task that required them to generate different levels of bite force at varying static gapes to receive a juice reward (Figure 1C and Supplementary Figure S1A). We recorded the bite force generated by the subjects while simultaneously recording neuronal responses from the OSMcx. Spiking activity of single neurons in MIo, SIo, and CMA was dynamically modulated during task performance; neurons exhibited increases and decreases in firing rates relative to the onset of bite force. Activity of some task-modulated neurons exhibited more robust tuning to gape than to bite force (compare Figures 2A–C, top vs. bottom row) while others showed activity that varied more with bite force than with gape (Figures 2D–F, bottom vs. top row).

### Encoding model

**Figure 3:**
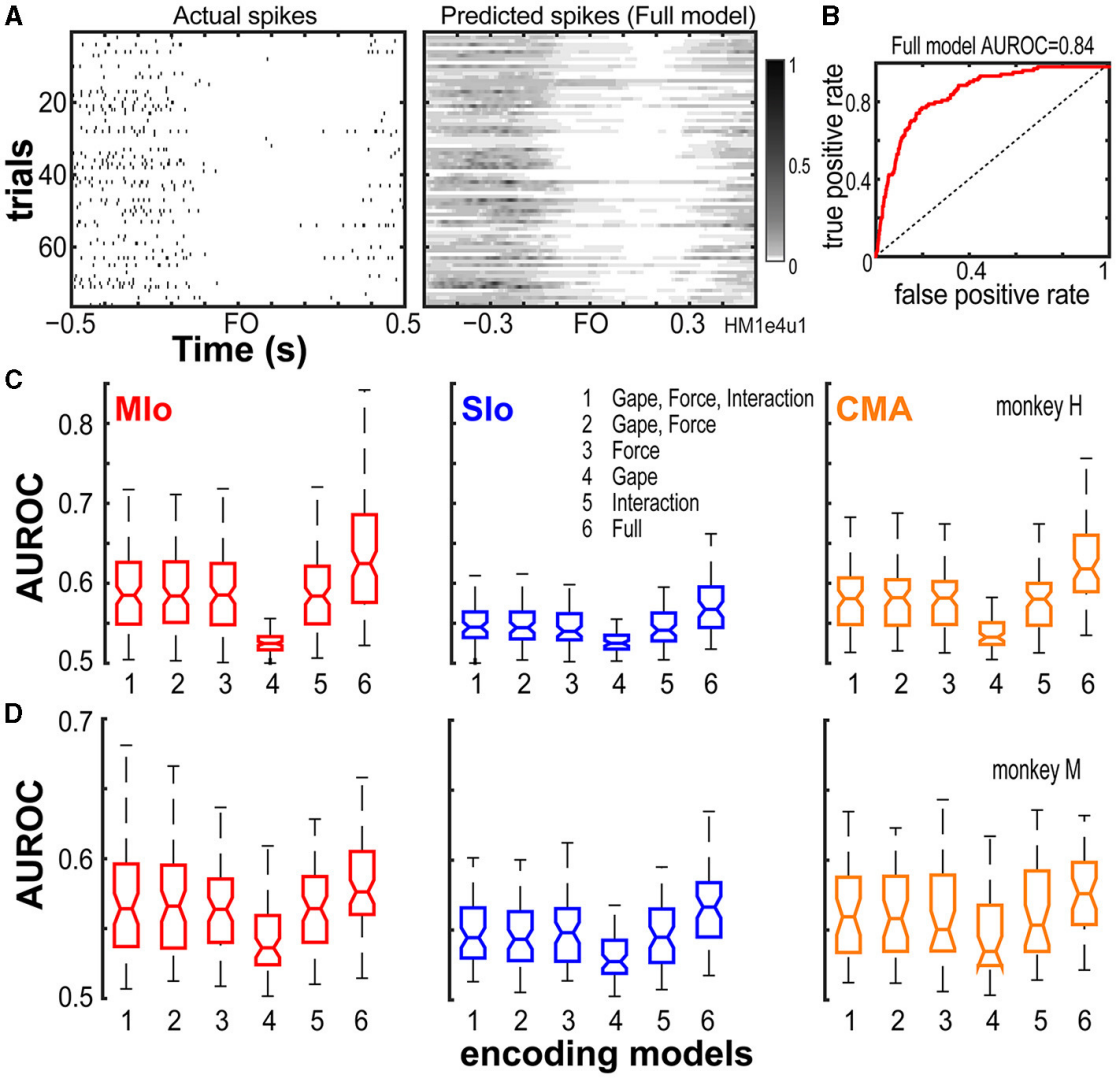
Performance of encoding models. **(A)** Actual firing rates of an example neuron vs. its predicted firing rates based on the full encoding model. **(B)** The full model's goodness of fit was quantified using the AUROC on a cross-validated test data for the neuron shown in **(A)**. Full model mean AUROC = 0.84. Dashed line denotes chance level. **(C, D)** Box plots of AUROC values from the population of neurons recorded from MIo, SIo and CMA (see Table 1 for *N*) are shown for each encoding model and animal. AUROC values are taken to be the mean across the 10-folds of cross-validation performed per neuron.

To determine the relative importance of bite force and static gape in predicting the firing of neurons and to compare encoding properties among these three cortical areas, we used GLM to predict the time-varying spiking activity of each neuron. The GLM approach allows us to measure how well a model predicts the mean spike count in a small time-window based on a set of input features that included extrinsic covariates (i.e., bite force, gape, and their interaction) as well as intrinsic, spike history covariates (Supplementary Figure S2, see Methods). Predictive power was assessed by computing the AUROCs on cross-validated test data. Figures 3A, B provides an example illustrating the actual firing rates of a MIo neuron, its predicted rates based on a full encoding model that included all input features, and the AUROC for a specific cross-validation of test trials for this neuron. The encoding model predicted the spiking activity of this neuron with a mean AUROC value of 0.84 across all ten runs of cross-validated test trials.

We then compared the predictive power (using AUROC) of a full GLM model having all covariates with reduced models having only a subset of covariates. On average, we found that all input features used in all encoding models, full or reduced, were able to predict spiking activity of most neurons in MIo, SIo, and CMA significantly better than chance (Wilcoxon signed-rank test, highest *p-*values given for all models used, H: MIo: *p* < 4x10^−20^; SIo: *p* < 1x10^−11^; CMA: *p* < 8x10^−14^; M: MIo: *p* < 2x10^−12^; SIo: *p* < 2x10^−11^; CMA: *p* < 5x10^−8^). However, the separate and combined ability of bite force, gape, and spike histories to predict the spiking of neurons differed; AUROCs were significantly different across the various encoding models, with the full encoding model showing the best performance and the gape-only model exhibiting the poorest performance (Figures 3C, D, Kruskal-Wallis test, H: MIo: *p* = 4x10^−44^; SIo: *p* = 5x10^−14^; CMA: *p* = 4x10^−25^; M: MIo: *p* = 2x10^−8^; SIo: *p* = 6x10^−9^; CMA: *p* = 0.003).

**Figure 4:**
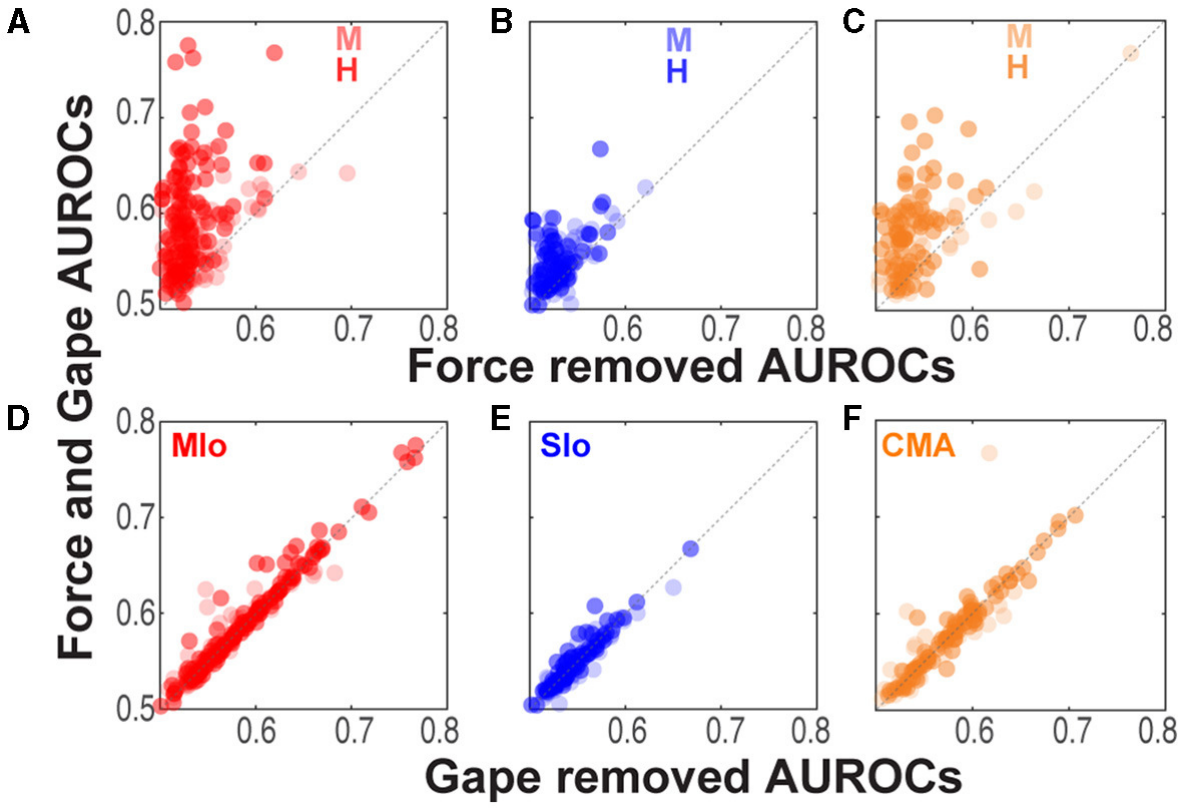
Comparison of model performance at an individual neuron level. **(A–C)** Relation between AUROCs of the joint force and gape model vs. the reduced model when force was removed. Each circle corresponds to a neuron's AUROCs. Shown for each animal and for MIo, SIo, and CMA, respectively (see Table 1 for *N*). AUROCs above the unity line (dashed line) denotes higher AUROCs in the joint vs. reduced model. **(D–F)** As in **(A–C)** but comparing AUROCs of the reduced model of when gape was removed.

We then sought to determine the relative importance of an input feature by comparing the performance of encoding models with and without the input feature in question. If an input feature contributes significantly to an encoding model, we would expect a model not to perform as well when that feature was removed. Figure 4 illustrates the degree of degradation of the predictive ability of an encoding model when either force or gape was removed by plotting each neuron's AUROC against the encoding model that included both gape and force (intrinsic covariates were not included in the model for this analysis). When removing the force feature, a majority of neurons clustered above the unity line due to significantly higher AUROCs in the combined force and gape model, indicating that excluding force from the encoding model degraded the model's predictive ability (Figures 4A–C, Wilcoxon signed-rank test, H: MIo: *p* < 5x10^−20^; SIo: *p* = 2x10^−9^; CMA: *p* = 6x10^−12^; M: MIo: *p* = 1x10^−8^; SIo: *p* = 1x10^−6^; CMA: *p* = 0.002). This was not the case when gape was removed, as shown in most neurons clustering along the unity line (Figures 4D–F, Wilcoxon signed-rank test, both subjects, all areas, *p* > 0.10). Thus, bite force was a more accurate predictor of spiking activity than gape. It may be argued that the difference in the predictive value of bite force and gape could be attributed to having bite force as a time-varying parameter of 8 features (i.e., 1,000 ms, 4-ms bins, 8 lags), while gape was given as a single, constant feature for each time bin. To verify this, we ran the GLM analysis using only one bite force value recorded at 100 ms after force onset and spike activity at one of six time points after force onset (60, 80, 100, 120, 140, and 160 ms). Altogether, we built six GLM models per neuron. We found that in 3–4 out of 6 time points evaluated, there was a significant degradation of prediction accuracy when force was removed and not when gape was removed in subject H (Kruskal-Wallis, MIo and SIo: *p* < 0.05) and in subject M (Kruskal-Wallis, CMA: *p* < 0.05). When not statistically significant, the trend was toward a higher predictive value of force vs. gape. Thus, the higher predictive ability of force was not completely biased by the way extrinsic features were used in the encoding model, suggesting that predictive power might be dependent on the movement parameter that drove successful performance of the task at hand.

We also considered the possibility that the interaction between gape and force might contribute significantly to model performance beyond the combined contribution of gape and bite force. However, our results did not show any evidence for this; the model that included force, gape, and their interaction performed similarly to a model that excluded their interaction (Figures 3C, D, compare 1 vs. 2, Wilcoxon signed-rank test, both subjects, all areas, *p* > 0.10). Lastly, the full encoding model, that included force, gape, their interaction, and spike history, outperformed encoding models that did not include spike histories (Wilcoxon Paired sign rank test, H: MIo: *p* = 5x10^−22^; SIo: *p* = 8x10^−12^; CMA: *p* = 2x10^−14^; M: MIo: *p* = 3x10^−12^; SIo: *p* = 8x10^−12^; CMA: *p* = 1x10^−7^).

While bite force accounted for most of the information that reduced encoding models used to predict spiking of individual neurons, the full encoding model that includes all input features (i.e., spike history, bite force, gape, and the interaction between them) outperformed all other reduced encoding models. Notwithstanding, the results indicated that each input feature carried distinct information capable of predicting spiking activity of neurons in all three areas of OSMcx as shown by reduced encoding models performing above chance level.

### Distribution of neurons encoding bite force or gape

**Figure 5:**
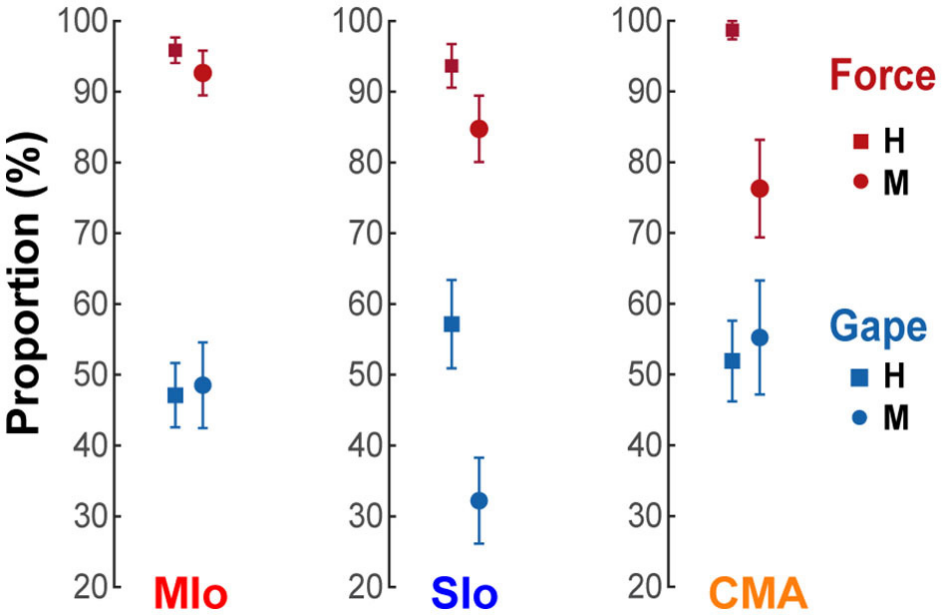
Proportion of neurons encoding bite force or gape. Proportion of neurons with AUROCs that were significantly higher than chance level in the reduced force and gape models. Only neurons whose models converged in at least 6 folds of cross-validated test data were included. Shown for each cortical area and subject [MIo: *N*_(forceonly, gapeonly)_ = (H:116/121 57/121; M:63/68 33/68); SIo: *N* = (H:59/63 36/63; M:50/59 19/59); CMA: *N* = (H:76/77 40/77; M:29/38 21/38)]. Error bars indicate ±1 SEM (based on a binomial distribution assumption).

We then evaluated whether there was any difference in the proportion of neurons encoding bite force compared with neurons encoding gape in the three studied areas of OSMcx. We identified “force-” or “gape-related” neurons as neurons whose AUROCs in the force or gape only encoding model, respectively, were significantly higher than chance level (Wilcoxon signed-rank test, *p*<*0.05*). Force-related neurons, comprising a mean of 91% (SE 4%) of the recorded neuronal population across all areas and animals, were predominant over gape-related neurons (58%, SE 4%) (Figure 5, *X*^2^ test, H: MIo: *p* = 1x10^−16^; SIo: *p* = 5x10^−6^; CMA: *p* = 6x10^−11^; M: MIo: *p* < 5x10^−8^; SIo: *p* < 2x10^−8^; CMA: *p* < 0.09). There were no significant differences in the proportion of either force- or gape-related neurons between any two cortical areas (*Chi-squared* test, significance at *p*<*0.017* after correction for multiple comparisons, i.e., MIo vs. SIo; MIo vs. CMA; SIo vs. CMA, H: all *p* > 0.10; M: all *p* > 0.04).

### Comparison of encoding model performance across cortical areas

**Figure 6:**
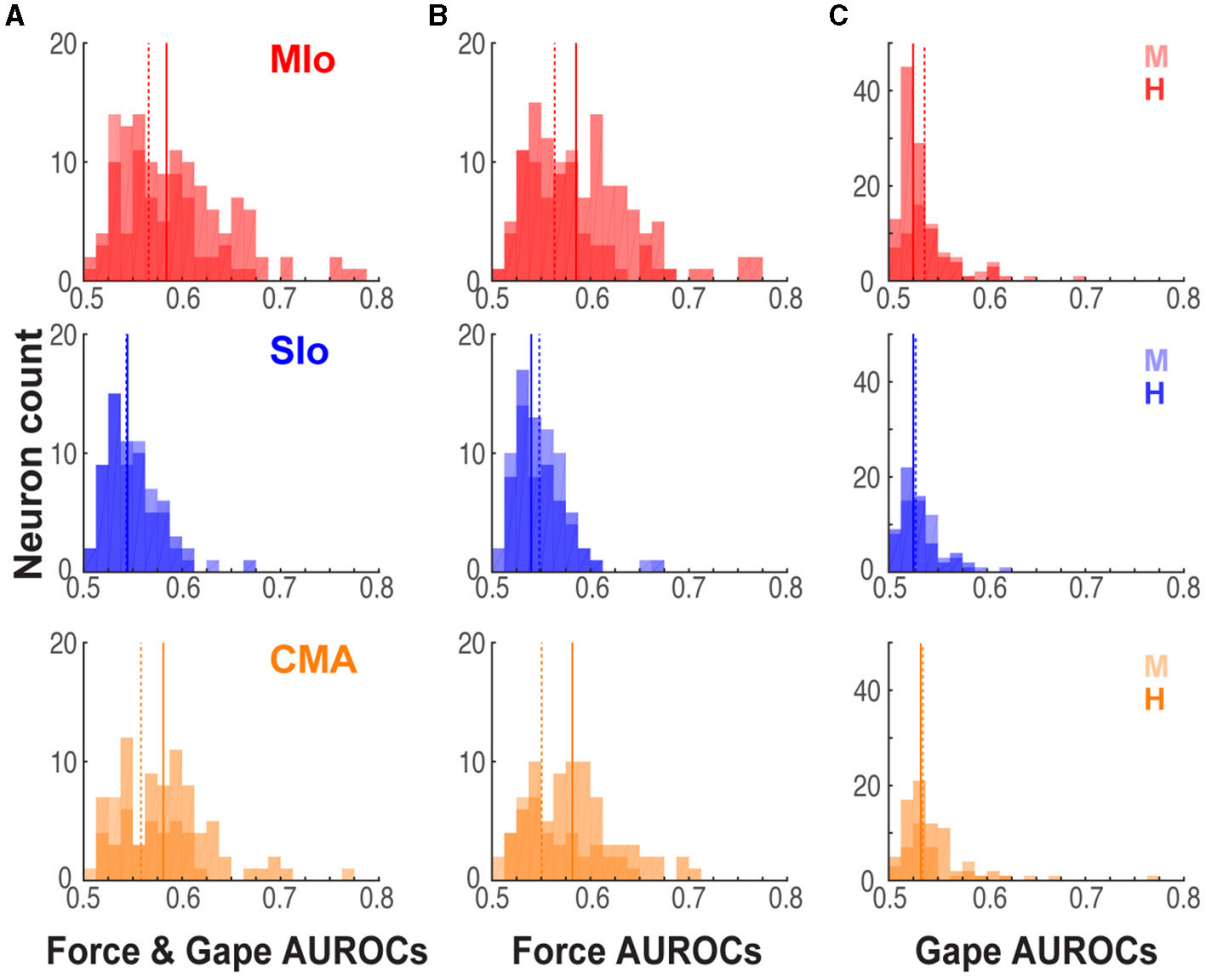
Performance of encoding models differed across cortical areas. **(A)** Distribution of significant AUROCs for the joint force and gape encoding model in MIo, SIo, and CMA, shown separately for each animal [MIo: *N*(H,M) = (121; 68); SIo: *N* = (63; 59); CMA: *N* = (77; 38)]. Solid and dashed lines denote median AUROCs for subjects H and M, respectively. **(B, C)** As in **(A)** for AUROCs for the reduced force and gape models [MIo: *N*(H,M), (force only, gape only) = (116 57; 63 33); SIo: *N* = (59 36; 50 19); CMA: *N* = (76 40; 29 21)].

To determine whether MIo, SIo, and CMA differ in the encoding of bite force and gape, we evaluated differences in the predictive ability of encoding models that included force only, or gape only, or both force and gape across the three areas. Using models with bite force and gape, we found a main effect of cortical area, where the activity of MIo was predicted better than the activity in SIo in both animals (Figure 6A, Kruskal-Wallis test, H: *p* = 6x10^−9^; M: *p* = 0.0007, *post-hoc paired comparison with Bonferroni correction MIo vs. SIo, H: p* < 0.0001, M: *p* < 0.001) but not any better than activity in CMA (Figure 6A, *post-hoc MIo vs. CMA, p* > 0.10). When comparing encoding models that had bite force as the only predictor for spiking, the predictive ability of force in MIo was better than SIo but not any better than CMA (Figure 6B, Kruskal-Wallis test, H: *p* = 5x10^−10^ *post-hoc MIo vs. SIo, CMA vs. SIo, p* < 0.0001; M: *p* = 0.017, *post-hoc MIo vs. SIo, p* < 0.05, *CMA vs. SIo, p* > 0.10; both subjects: *post-hoc MIo vs. CMA, p* > 0.10). When encoding models had gape as the only predictor, a main effect of cortical area was observed in subject H where the model's predictive performance was best in CMA (Figure 6C, Kruskal-Wallis test, H: *p* = 0.0016, *post-hoc CMA vs. MIo, p* < 0.01, *CMA vs. SIo, p* < 0.05). No significant differences between cortical areas were found in subject M (Kruskal-Wallis test, *p* = 0.055).

### Relative importance of temporal lags of bite force

**Figure 7:**
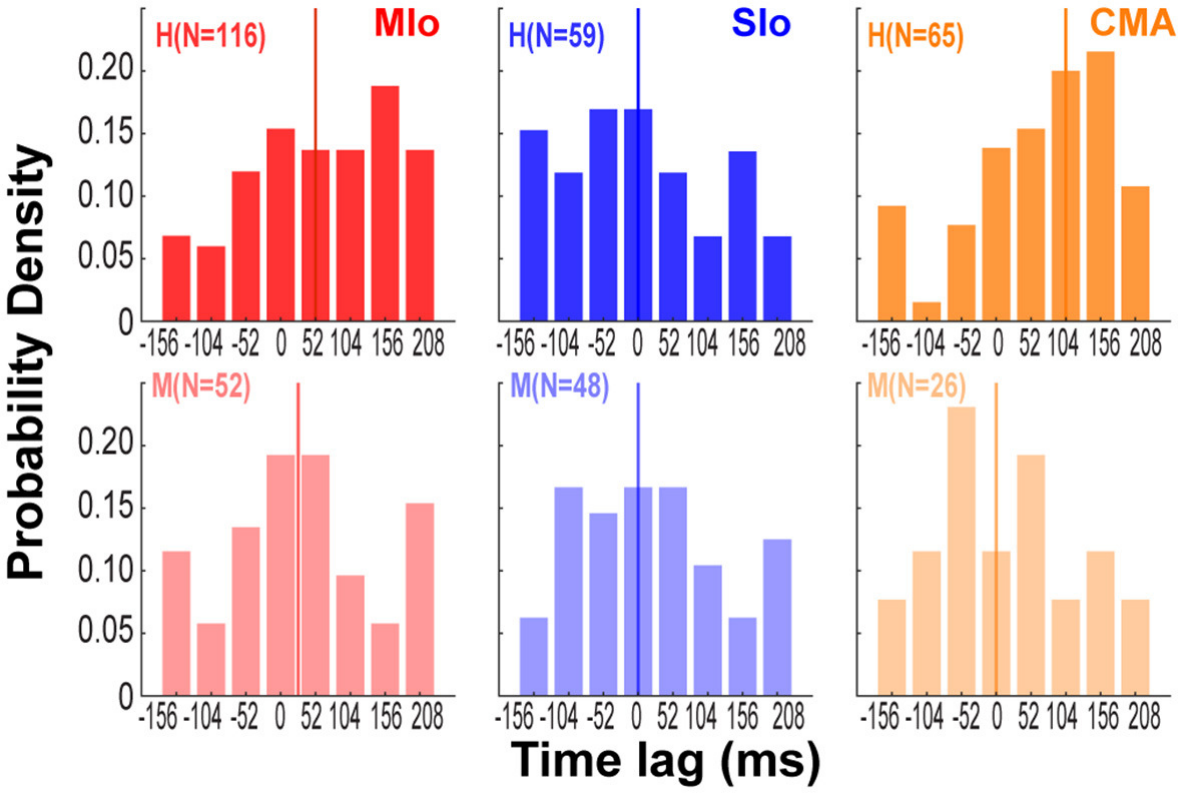
Preferred temporal lags of bite force differed across cortical areas. Distribution across neurons of time lags associated with maximum coefficient for force in each cortical area and subject. Colored vertical lines indicate median values of each distribution [MIo: *N*(H,M) = (116; 52); SIo: *N* = (59; 48); CMA: *N* = (65; 26)].

Since we found that bite force is more strongly encoded than gape in all three cortical areas, we next evaluated the impact of temporal lags in bite force (from −156 to 208 ms relative to spiking) on predicting spiking of neurons in relation to bite force. For this analysis, we used the absolute values of the β coefficients of the temporal lags of bite force from the encoding model with force as the only predictor. Only β coefficients that were significantly different from zero were included (*t*-Test, *p* < 0.05). For each neuron, we found the time lag that was associated with the largest β coefficient and computed the distribution of time lags across neurons for each cortical area (Figure 7). Although the distributions of time lags were quite broad, there were important differences across cortical areas. The median time lags for MIo were 52 and 26 ms for subjects H and M, respectively, indicating that force lagged spiking and consistent with the view that MIo drives force generation. In contrast, the median time lags for SIo were 0 ms for both subjects. In CMA, the results were inconsistent across animals suggesting a more heterogeneous temporal relationship between force generation and neural responses (H:104 ms, M: 0 ms).

### Relative importance of spike history timescale to spiking activity

The β coefficients for the most immediate spike history (16 ms) were higher than β coefficients for spikes that occurred further in the past (44 or 108 ms) for all areas in both animals (Supplementary Figures S3A, B, Kruskal- Wallis, H: all *p* < 2x10^−5^, M: all *p* < 3x10^−5^, *post-hoc* multiple comparison with Bonferroni correction, *p* < 0.05). This indicates that immediate past history outweighs the other timescales in the ability to predict spiking of neurons in OSMcx.

### Population encoding of behavioral parameters

**Figure 8:**
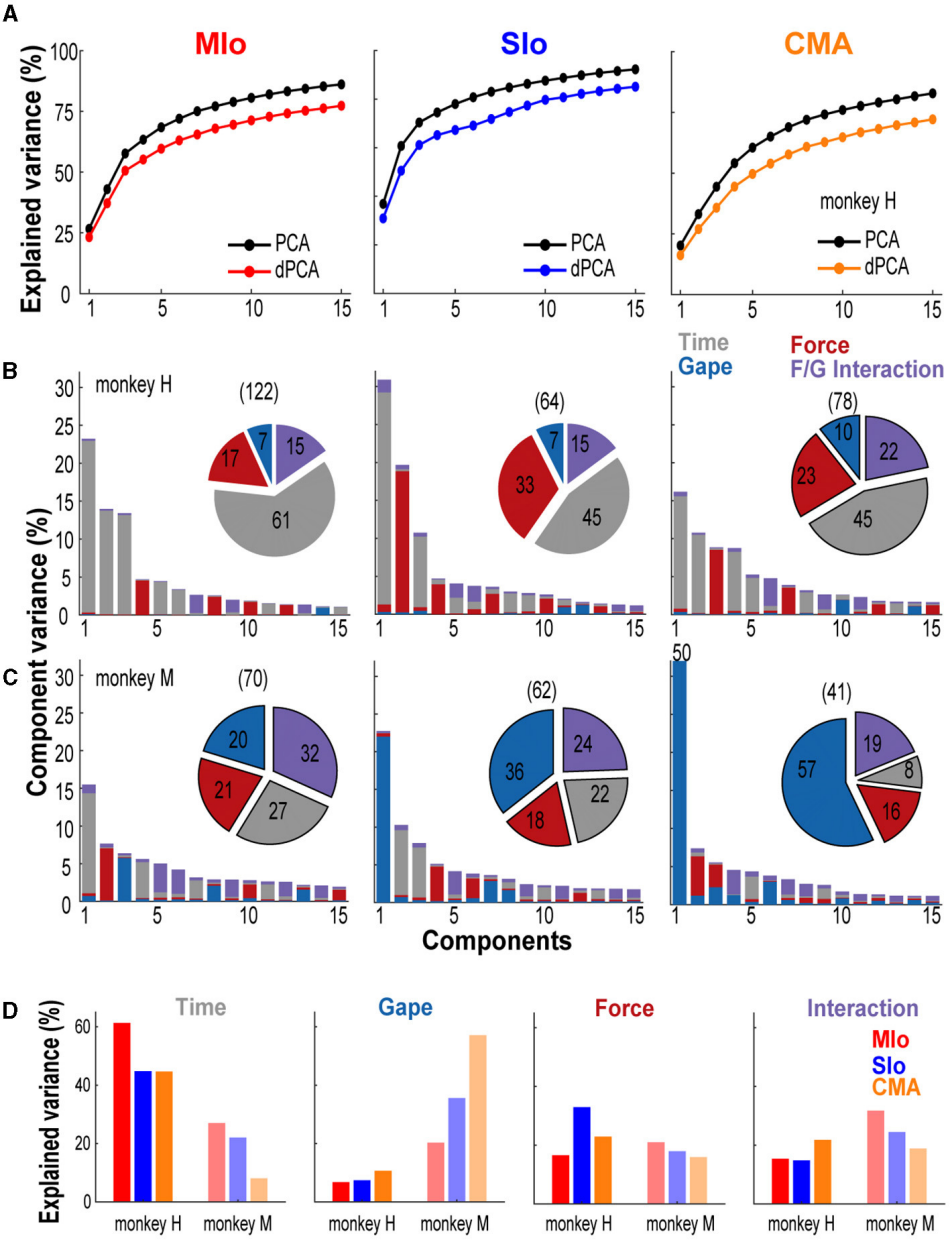
Neural variance accounted for by demixed principal components. **(A)** Comparison of cumulative variance explained by PCA and dPCA. Shown for the first 15 components and for each cortical area separately. Data from subject H. **(B, C)** Bar graphs illustrating the proportion of variance accounted for by each task parameter (color) corresponding to individual dPCs in subjects H and M, respectively. Single-colored bars depict complete demixing. Pie chart illustrates the proportion of variance (%) explained by task parameters. Numbers in parenthesis denote total number of neurons used in the analyses. Shown for each cortical area. **(D)** Across-area comparison of the proportion of variance (%) explained by each task parameter shown for both subjects.

While encoding models using GLMs showed that bite force more accurately predicted the individual neuron's spiking than did gape, our simultaneous, multi-site recordings allowed us to examine how activity at the population level in MIo, SIo, and CMA represents bite force and gape. Thus, we investigated how activity of neuronal populations in MIo, SIo, and CMA might distinguish between these behavioral parameters. Here, we used a latent variable model, dPCA (Kobak et al., 2016) to decompose the dependencies of the population activity into a task-independent parameter of *time* (for activity related to the progression through the behavioral trial), and task-dependent parameters of *bite force* and *gape*, and the *interaction* between them. This approach was used to capture the structure of the population-wide activity patterns, i.e., latent activity. Figure 8 illustrates the cumulative variance in the population signal accounted for by demixed principal components (dPCs), i.e., neural modes, and the variance accounted for by individual task parameters for each cortical area. Over 70% of the variance was accounted for by 7–13 dPCs in subject H (Figure 8A) and 6–20 dPCs in subject M. The first five dPCs showed very good demixing of task parameters as most of the component variance was explained by a single task parameter, such as the time-related activity for the first dPC or bite force for the second dPC in SIo (Figure 8B center).

The task parameter that explained most of the variance differed across cortical areas; for both animals, the variance explained by the condition-independent component was highest in MIo. We also observed that the time-related activity in all three areas was very prominent in subject H (45–60%) compared to 8–27% in subject M (pie charts in Figures 8B–D). The variance explained by static gape was higher in CMA than in SIo and in MIo for both animals (Figure 8D). The difference across cortical areas was pronounced in subject M, where randomized design was used, as the explained variance in CMA was nearly triple that in MIo (MIo: 20%, SIo: 36%, CMA: 57%). Variances accounted for by gape in all three areas were substantially higher in subject M (20–57%) than in subject H (7–10%). The variance accounted for by bite force was highest in SIo for subject H, which was almost twice the total variance explained by bite force in MIo (Figure 8D). Lastly, the variance explained by interaction between gape and force in all cortical areas ranged between 15–32% across subjects, suggesting a relative importance of the coordinated control of these two parameters (Figure 8D). Similar results were also found when dPCA was performed on a subset of trials and using two other datasets (Supplementary Figure S4).

**Figure 9:**
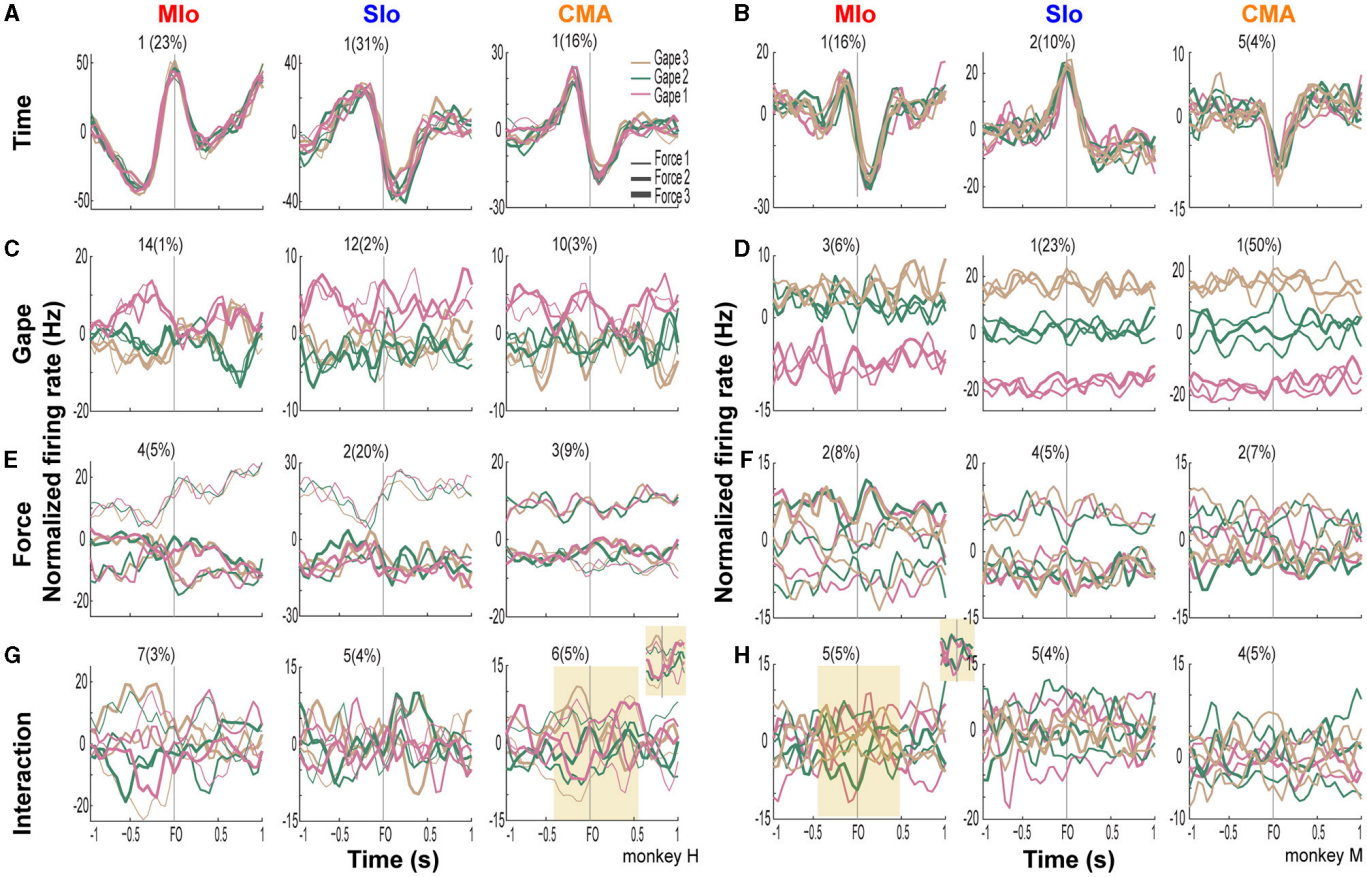
Latent activity of leading demixed principal components of task parameters. **(A, B)** Projections of population activity onto the leading dPCs of condition-independent parameter of time in subjects H and M, respectively. Each subplot shows 9 lines corresponding to 9 trial types for each cortical area. The component number and corresponding explained variance is shown above each subplot. **(C–H)** As in A-B, for gape, bite force, and the interaction between gape and force. Insets show zoomed periods around force onset when activity related to trial types show clear separation or overlap.

In all cortical areas, latent activity for static gape and bite force exhibited separation by trial types. Figure 9 illustrates the linear projections of population latent activity in MIo, SIo, and CMA corresponding to dPCs with the highest explained variance for each of the task parameters. Latent activity for gape distances exhibited varying degrees of separation across areas (Figures 9C, D). In subject M, where higher explained variances for static gape were observed in SIo and CMA, latent activity was well-separated by gape levels and exhibited increasing activity with increasing gape levels. This was not observed in subject H where explained variances for gape were low across all cortical areas. Activity of the lead dPC in MIo separated only between minimal gape and medium/wide gape (Figure 9D), as also found in all cortical areas in subject H (Figure 9C). Across areas and animals, latent activities corresponding to different bite force levels were also separated, although tuning to bite force levels was observed at varying strength and times relative to force onset (Figures 9E, F). Separation of latent activities by force levels was more pronounced in MIo, with maximal separation occurring at force onset in both subjects. Both SIo and CMA exhibited distinct activities between two force levels only. Lastly, latent activity of dPCs corresponding to the interaction between gape and bite force showed varying degrees of separation at different times relative to force onset (Figures 9G, H). For example, trial types were more separated around 0.3 s prior to force onset but became more overlapping after force onset (Figure 9G inset). The activity of interaction components appeared complex, having distinct and overlapping activity patterns for certain gape-force combinations; low bite force generated at gape distances 1 and 2 had activity patterns opposite to high bite force applied at these gape distances (Figure 9H inset). A subset of neurons that carry both gape and bite force information may underlie the coordination between these features.

**Figure 10:**
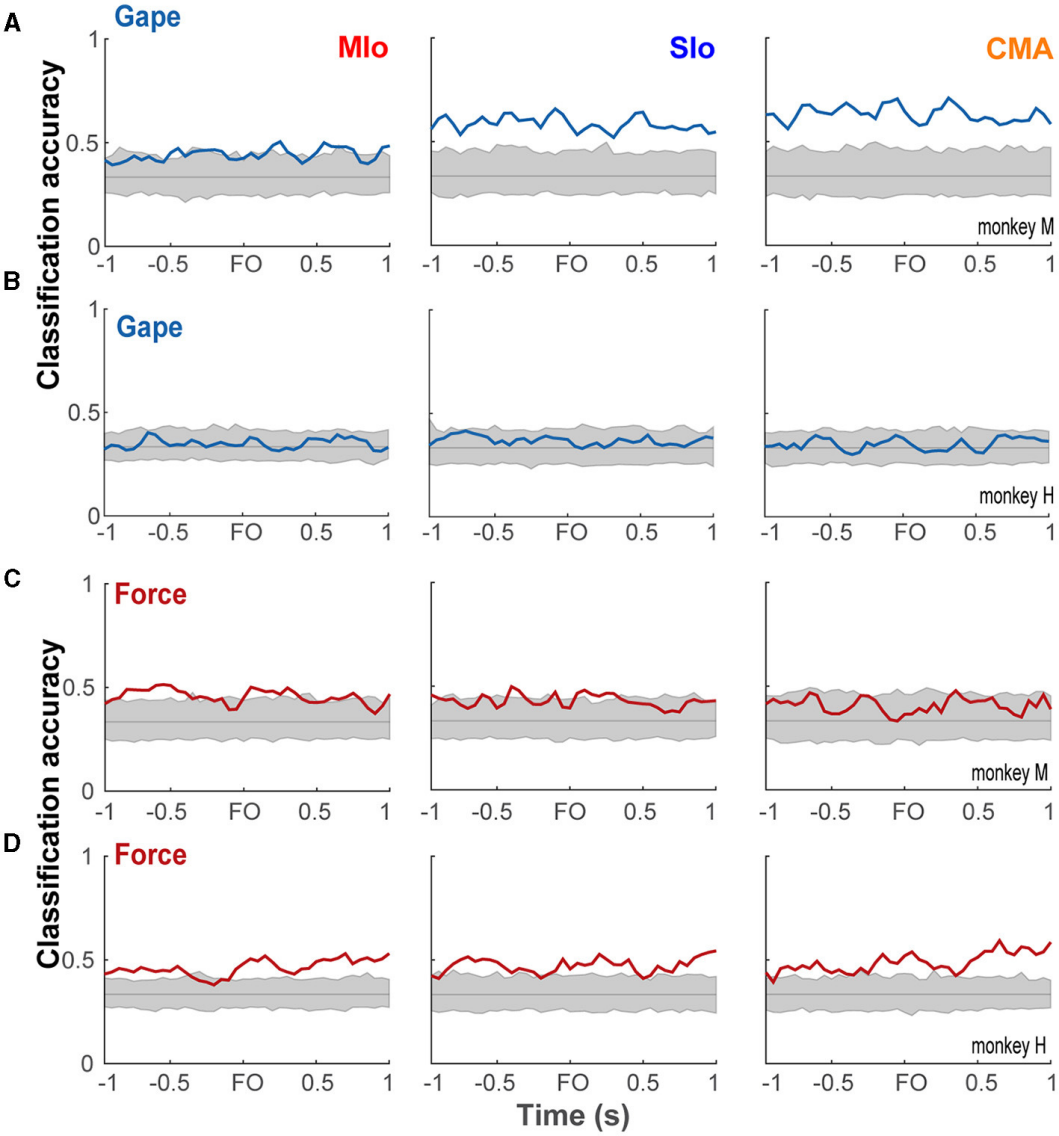
Classification performance of bite force and gape components. **(A, B)** Classification accuracies (blue line) of linear classifiers given by the first gape dPC in subject M and H, respectively, shown for each OSMCx area. Shaded gray area correspond to the distribution of classification accuracies expected by chance (solid gray line) as estimated by 500 iterations of shuffling procedure. **(B, C)** As in A but using the first bite force dPC in subjects M and H, respectively.

The latent activity patterns of dPCs provided useful information about the modulation of population activity relative to behavioral events and task parameters, motivating us to evaluate the performance of dPCs in decoding gape and force at a single-trial level. Using the first dPC for each task parameter as a fixed linear classifier, we evaluated the accuracy of classifying gape and force levels. Classification of gapes was significant in subject M only. Figure 10A shows significant classification of gapes ±1 s relative to force onset in SIo and CMA and in shorter periods in MIo. In the case of bite force, classification accuracy was significant in all areas for subject H for most periods and in MIo and SIo for shorter periods in subject M (Figures 10B, C). Classification accuracy using interaction components did not show any time period with significant performance.

## Discussion

In this study, we investigated how individual neurons and populations of neurons in multiple regions of OSMcx differentially encode bite force and gape when subjects generated different levels of bite force at varying static gapes during a biting task. Our results add new insights to our understanding of sensorimotor control of oromotor behavior by showing that in the biting task, (1) the activity of individual neurons in MIo, SIo, and CMA were strongly tuned to bite force compared to static gape, (2) the simultaneous encoding of bite force and gape was better in MIo and CMA neurons, compared to SIo neurons, and (3) the population activity revealed robust tuning to both static gape and its interaction with bite force, that was not evident from the single-neuron encoding model.

### Spiking activity of individual neurons in OSMcx is better predicted by bite force than by gape

Past studies have demonstrated that MIo and SIo neurons modulate their activity to changes in bite force, jaw position, and movement (Lund and Lamarre, 1974; Hoffman and Luschei, 1980; Murray and Sessle, 1992a; Lin et al., 1994). In the current study, simultaneous recording in MIo, SIo, and CMA while subjects performed a biting task at varied combinations of gape and bite force levels allowed us to determine the relative importance of these task parameters in predicting the firing of neurons and to explore how these areas might assume diverse roles in the cortical control of a functionally important oromotor behavior. We demonstrated that when subjects performed a biting task, neurons in each of the three areas encoded bite force more strongly than gape (see Figure 4) and that the force-related neurons were more predominant than the gape-related neurons. This was true regardless of the sequence used in presenting trials (i.e., blocked vs. randomized gapes). A possible explanation is that a larger portion of the cortical space was used because of a higher complexity in the task that involved dynamic control of varying bite force levels for successful performance, whereas gape remained static and was not actively controlled by the subject to receive the reward. The different control requirements for bite force and gape influence how muscles are activated, and which sensory information is more relevant. Because the task does not require voluntary control of jaw depression (opening), activation of jaw depressors (anterior digastric, mylohyoid, and inferior head of the lateral pterygoid) is not expected because the lower jaw is passively depressed to a predetermined static gape prior to the generation of the required bite force. Instead, jaw elevators (masseter, temporalis, medial pterygoid, and superior head of the lateral pterygoid) are expected to be activated to produce the required force level that varies during the trial. In this scenario, OSMcx neurons may be involved in the selective excitation of jaw elevators and inhibition of jaw depressors for the generation of varying bite forces (Hoffman and Luschei, 1980; Moustafa et al., 1994). The OSMcx receives information about the changing magnitudes of bite force and gape, in part via thalamus. This information is derived from neurons in the trigeminal mesencephalic nucleus and trigeminal ganglion that innervate muscle spindles of masticatory muscles or the mechanoreceptors of the periodontal ligaments of the teeth and the temporomandibular joint (Larson et al., 1981, 1983; Huang et al., 1989b; Lund and Kolta, 2006; Trulsson, 2007; Wang and May, 2008). As sensory information on the position of the lower jaw remains unchanged during bite force generation in this task, the most critical sensory information for successful task performance is the magnitude of applied bite force. In a task that requires changes in jaw position, such as in chewing, OSMcx neurons may show more robust tuning to gape as both muscles and afferents are activated during dynamic gape changes.

The results have relevance to the understanding of sensorimotor principles that span multiple domains, for example, lifting a load at varying degrees of wrist/elbow extension (isotonic) and subsequent isometric contraction to hold the load. Indeed, our findings are consistent with previous findings reported in a limb study in which static effects (i.e., encoding of hand position when the hand is held static at the different targets) were compared to dynamic effects (when the arm is moving to the targets) on neuronal modulation in the motor cortex and area five of rhesus macaques (Georgopoulos and Massey, 1985). In that study, they showed that modulation of neurons during dynamic movement was larger than that which would be expected if the hand were positioned statically at each point in the path from the center target to peripheral target. In our case, jaw gape is a static variable whereas bite force is dynamic.

### Gape-related activity is better represented at the population level

Linear projections of population activity (i.e., latent activity) using dPCA in MIo, SIo, and CMA revealed robust tuning to gape and its interaction with bite force that was not apparent at the level of individual neurons. Indeed, in subject M where gape varied randomly trial-to-trial, the neural variance accounted for by gape was double to triple that of bite force (see Figure 8D) and single-trial decoding of gape distances was significantly higher than chance (see Figure 10A), notwithstanding the poor predictive of single neuron encoding models with only gape as the input feature (see Figure 3D). It is of interest that the temporal dynamics of the gape-related population activity revealed a cyclic or oscillatory pattern (Figures 9C, D). These oscillations may be related to cyclic, short-range jaw depression-elevation for bite force generation, throughout the trial exhibited by subject M. Alternatively, the cyclic pattern may be related to non-movement related factors such as posture maintenance involving coactivation or reciprocal inhibition of jaw-closing and jaw-opening muscles, similar to postural control processes during limb movements (Humphrey and Reed, 1983; Lacquaniti et al., 1997; Feldman, 2016; Heming et al., 2016). In this context, the gape-related activity of OSMcx neurons may set the postural state of the jaw to the appropriate postural background for movement based on the expected sensory and motor consequences of the interaction of jaw dynamics and environmental factors on which bite force is generated and fine-tuned to meet task demands. Thus, sensory and motor systems are prepared for upcoming information from the external environment as well as from internal biomechanical changes. Similarly, randomization of gape on a trial-to-trial basis may have increased the demand for attention and reduced the predictability of task parameters, thus, diminished the ability to anticipate the appropriate sensorimotor response.

One may ask how does the accurate classification of gape emerge in population coding when individual neurons carry low information about gape? While the predictive ability of gape was weaker than bite force across individual neurons, some single neurons in MIo, SIo, and CMA exhibited significant modulation of spiking activity as a function of gape, as shown in the PETHs in Figures 2A–C and in AUROCs >0.6 in Figures 4A–C, especially in subject M. Encoding models of single neuron responses such as GLM as used in our study did not account for the influence of other neurons' spiking activity/history on predicting a specific neuron's spiking activity. In contrast, the decoding method in dPCA uses the decoding axes of the first dPCs as linear classifiers to decode gape (or force), thus, taking in the weighted combinations of the activity of all recorded neurons in a specific region, which are also influenced by neurons from other regions. Population activity analysis using dimensionality reduction techniques can capture the underlying network connectivity as patterns of covariation across connected neurons (Cunningham and Yu, 2014; Gallego et al., 2017). Thus, the low information on gape at the level of individual neurons vis-à-vis the accurate classification of gape at the population level suggests that control of gape requires a coordinated response from connected neurons.

### Neuronal population encoding of task parameters reveals possible context-dependent modulation

Here we showed that the latent activity of populations of neurons in MIo, SIo, and CMA discriminated between task parameters, consistent with previous findings in other brain regions (Kobak et al., 2016; Gallego et al., 2018). dPCA also allowed us to capture features of population activity that were common or diverse. All three OSMCx areas shared neural modes (dPCs) with comparable task-independent, time-varying activation patterns while accounting for population variance in varying proportions. In our study, the time-related activity was very prominent in subject H (45–60%) compared to 8–27% in subject M. This was not surprising as other studies reported similar values ranging from 26 to 86% (see Figures 3, 4 in Kobak et al., 2016 and Figure 4 in Gallego et al., 2018). Moreover, we also found a substantial inter-subject difference in the variance explained by gape; in subject M, the explained variance for gape was more than double that observed in subject H. While slight variations in the implantation sites of the multielectrode arrays may be a contributing factor (Supplementary Figure S1), we speculate that this effect is minor as the proportions of force- and gape-related neurons were comparable between subjects. Other factors that we are unable to control or monitor, such as attention, motivation, or task proficiency, may exert influence and explain the different results between subjects. Alternatively, the higher explained variance for gape in subject M and explained variance for time in subject H may be attributed to the differences in task structure. The trial-to-trial randomization of gapes vs. blocked trials of single gapes may have a higher task complexity to regulate gape changes from trial-to-trial. If so, this would suggest that task context might adjust the contribution of relevant task parameters in determining the population activity, thus serving as population encoding of differing contextual information for similar movement topologies, i.e., generating bite force at varying gapes. The results are consistent with previous findings showing context-dependent modulation of cortical encoding during texture discrimination in task vs. no-task conditions and grasping behavior with regular vs. irregular ladder wheels (Jiang et al., 2018; Omlor et al., 2019).

### Diverse functions of orofacial cortical regions

Neuronal activity patterns, receptive field (RF) features, properties of evoked rhythmic jaw movements, and cortical effects of ablations or reversible cold blocks differ across these three areas of the OSMcx (Luschei and Goodwin, 1975; Huang et al., 1988, 1989a; Murray et al., 1991; Lin et al., 1993, 1998; Martin et al., 1999; Narita et al., 2002; Yamamura et al., 2002; Yao et al., 2002; Hatanaka et al., 2005; Arce et al., 2013; Arce-McShane et al., 2014, 2016). Our results demonstrate that MIo, SIo and CMA are all involved in the control of bite force and static gape but differ quantitatively in their representation of these two parameters. While individual neurons in all three cortical areas encoded bite force more strongly than static gape, MIo and CMA were better than SIo in predicting spiking activity based on bite force. The similarity between MIo and CMA may be related to anatomical overlap between borders of lateral MIo and CMA. The differences between cortical areas are unlikely to come from differences in RF properties as the RFs of neurons in all three areas are similar in having bilateral representations, although they are predominantly contralateral in SIo (Huang et al., 1988, 1989b; Hatanaka et al., 2005; Sessle et al., 2005). Thus, their difference may reflect differing functions with regards to motor- vs. sensory-related signals as well as density of network connections with other brain regions. For example, in addition to inputs from SIo, relevant sensory inputs also reach MIo and CMA via thalamo-cortical or cortico-cortical pathways. These findings point more to the role of SIo in modulating these types of behavior rather than generating them. The better representation of gape in CMA at the population level may be related to the involvement of CMA, which includes the lateral zone of MIo (Huang et al., 1989b; Martin et al., 1999; Hatanaka et al., 2005), in both rhythmic jaw movements as well as more elemental jaw-opening movements, all of which involve changes in gape. Moreover, the distinctive patterns of evoked rhythmic jaw movements described in previous studies suggest a role for distinctive masticatory patterns that can be attributed to input-output organization in these three OSMcx areas. Cortico-striatal and cortico-tegmental projections differ between MIo and CMA but have similar thalamo-cortical connections (Hatanaka et al., 2005). Further studies are required to determine whether the diverse functions of these three areas could be more pronounced during the different stages of feeding behavior wherein distinctive masticatory, tongue, and swallowing patterns are naturally generated.

## Limitations of the study

(1) Since the experimental paradigm is limited to static gape during an incisor biting task, our conclusions do not extend to cortical representations involved in biting behavior involving more posterior teeth or in chewing behavior that is characterized by active jaw movements that accompany bite force generation. Nevertheless, static gape does characterize certain forms of natural feeding, such as when one bites into a hard substance such as nuts or carrots, or other behaviors such as teeth clenching. While a full characterization of gape encoding would require dynamic jaw movements, this study provides an important first step in investigating simultaneous encoding of bite force and gape in three cortical areas and for future studies that involve complex and dynamic motor tasks encompassing transitions from static jaw postures to jaw movement. (2) The population results showed an enhanced encoding of static gape and its interaction with bite force in only one subject, which we attribute primarily to the randomization in the presentation of gape trials. Further studies are required to investigate the effect of the complexity of task requirements and trial presentation on the differential encoding of bite force and gape at the level of individual neurons and populations.

## Conclusion

Here, we investigated the cortical representations of bite force and gape by individual neurons and large populations of neurons across connected motor and somatosensory areas in OSMcx. We showed that bite force was more strongly encoded than gape by individual neurons in MIo, SIo, and CMA. Moreover, bite force was more effectively represented in motor vs. somatosensory cortices. At the population level, bite force and gape were distinguishable in the monkey trained with the randomized task. The results are important for understanding neurophysiological processes underlying biting and masticatory dysfunctions that may occur in aging, stroke, some pain states (e.g. temporomandibular disorders), and neurodegenerative diseases such as Parkinson's and Alzheimer's disease.

## Data availability statement

The raw data supporting the conclusions of this article will be made available by the authors, without undue reservation.

## Ethics statement

The animal study was approved by University of Chicago Animal Care and Use Committee. The study was conducted in accordance with the local legislation and institutional requirements.

## Author contributions

Conceptualization and writing—review and editing: FA-M, NH, CR, and BS. Data collection: FA-M and YR. Software, analysis, and writing—original draft: FA-M. All authors contributed to the article and approved the submitted version.
