# Supplementary material for: Multiple regions of sensorimotor cortex encode bite force and gape
Source: Front Syst Neurosci. 2023 Sep 22;17:1213279. doi: 10.3389/fnsys.2023.1213279 (PMC10556252; doi:10.3389/fnsys.2023.1213279)
Supplement: Supplementary file 1 [file Data_Sheet_1.docx]

Supplementary Material

Multiple Regions of Sensorimotor Cortex Encode Bite Force and Gape

Fritzie I. Arce-McShane^1,2*^, Barry J. Sessle^3^, Yasheshvini Ram^4^, Callum F. Ross^4#^, Nicholas G. Hatsopoulos^4#^

***** Corresponding Author: fritziea@uw.edu

# Supplementary Methods

***Behavioral task.*** The behavioral program was written using Matlab (Mathworks, Natick, MA) Toolbox Brain-computer interface to virtual reality (BCI2VR)(Bai et al., 2007). Behavioral event logs and timestamps were sent to the neural data acquisition system, Grapevine Neural Interface Processor (Ripple Neuro, Salt Lake City, UT). Force transducer (Micro Measurements, Raleigh, NC) analog signals were sampled at 1 KHz and stored using the neural data acquisition system. At the start of the recording session, monkeys were positioned such that their front teeth are pressed against the tip of the bite plates. The trial started with the movement of the bite plates to a specific gape. A cursor that represented the amplitude of the bite force appeared 1 sec after the bite plate reached the desired gape. After a random period between 0.75 to 1.25 s from the appearance of the cursor, the base target window appeared to cue the monkey to keep the cursor within the base target window by applying a low-level isometric bite force for a hold period of 0.3 s. Upon successful hold at the base target, the force target window appeared to signal the monkey to move the cursor into the force target window. Three force target levels were set accordingly to the animal’s comfortable bite force range while keeping the visual displays of the target the same. The size of the force target windows corresponded to a range of ±0.25 V from the required force level. To achieve success, monkey had to generate the required force within the allotted time (5 s) and to hold the force 0.1 s for monkey M and 0.05 s for monkey H. To indicate success, the force target window changed color and the monkey received a reward. We set an inter-trial interval of 3 s. For all analysis, we used trials where the subject was able to reach the target (H: 197/246 trials; M=129/183).

**References**

Bai, O., Lin, P., Vorbach, S., Li, J., Furlani, S., and Hallett, M. (2007). Exploration of computational methods for classification of movement intention during human voluntary movement from single trial EEG. *Clin. Neurophysiol.* 118, 2637–2655. doi: 10.1016/j.clinph.2007.08.025.
