# Supplementary figures and images for: Multiple regions of sensorimotor cortex encode bite force and gape
Source: Front Syst Neurosci. 2023 Sep 22;17:1213279. doi: 10.3389/fnsys.2023.1213279 (PMC10556252; doi:10.3389/fnsys.2023.1213279)

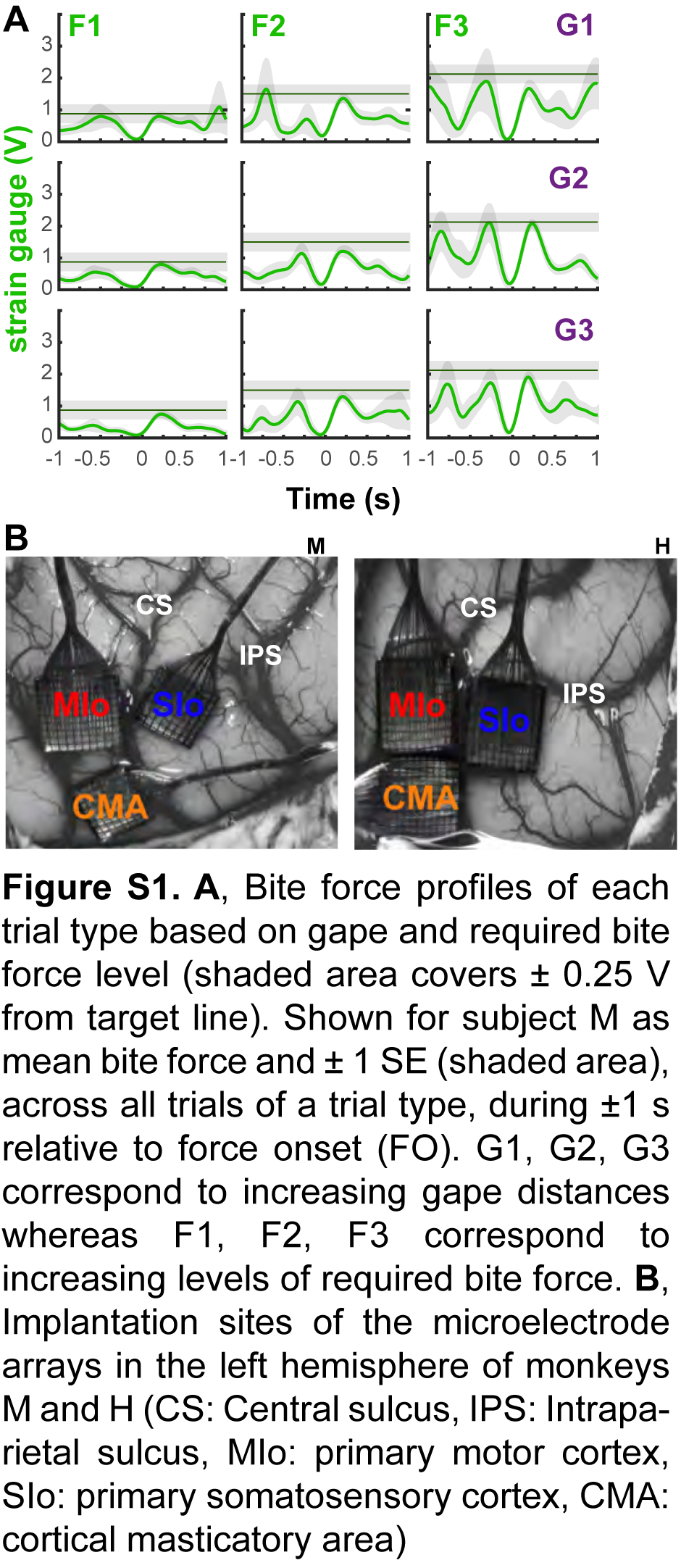

Supplement: Supplementary file 2 [file Image_1.tif]

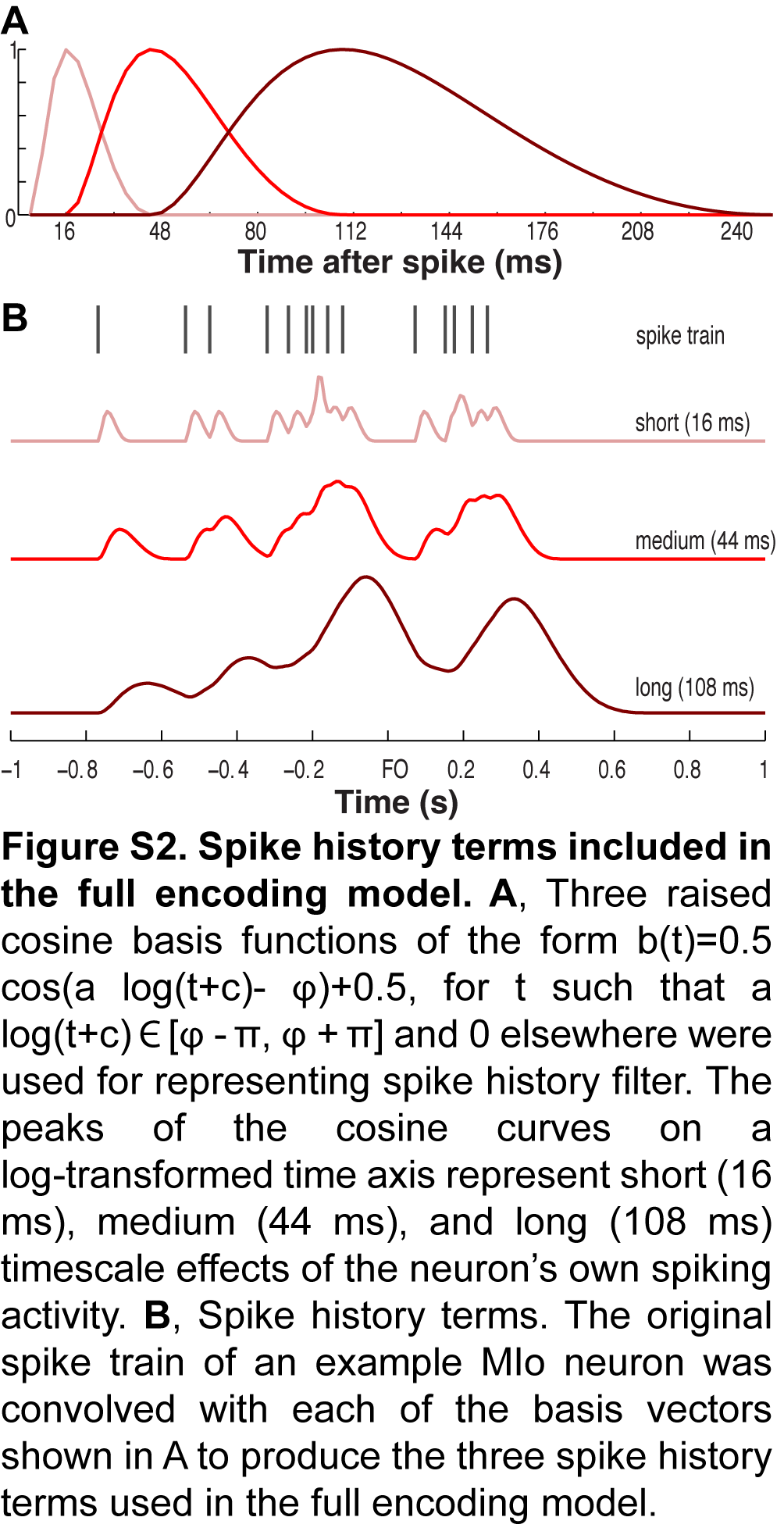

Supplement: Supplementary file 3 [file Image_2.tif]

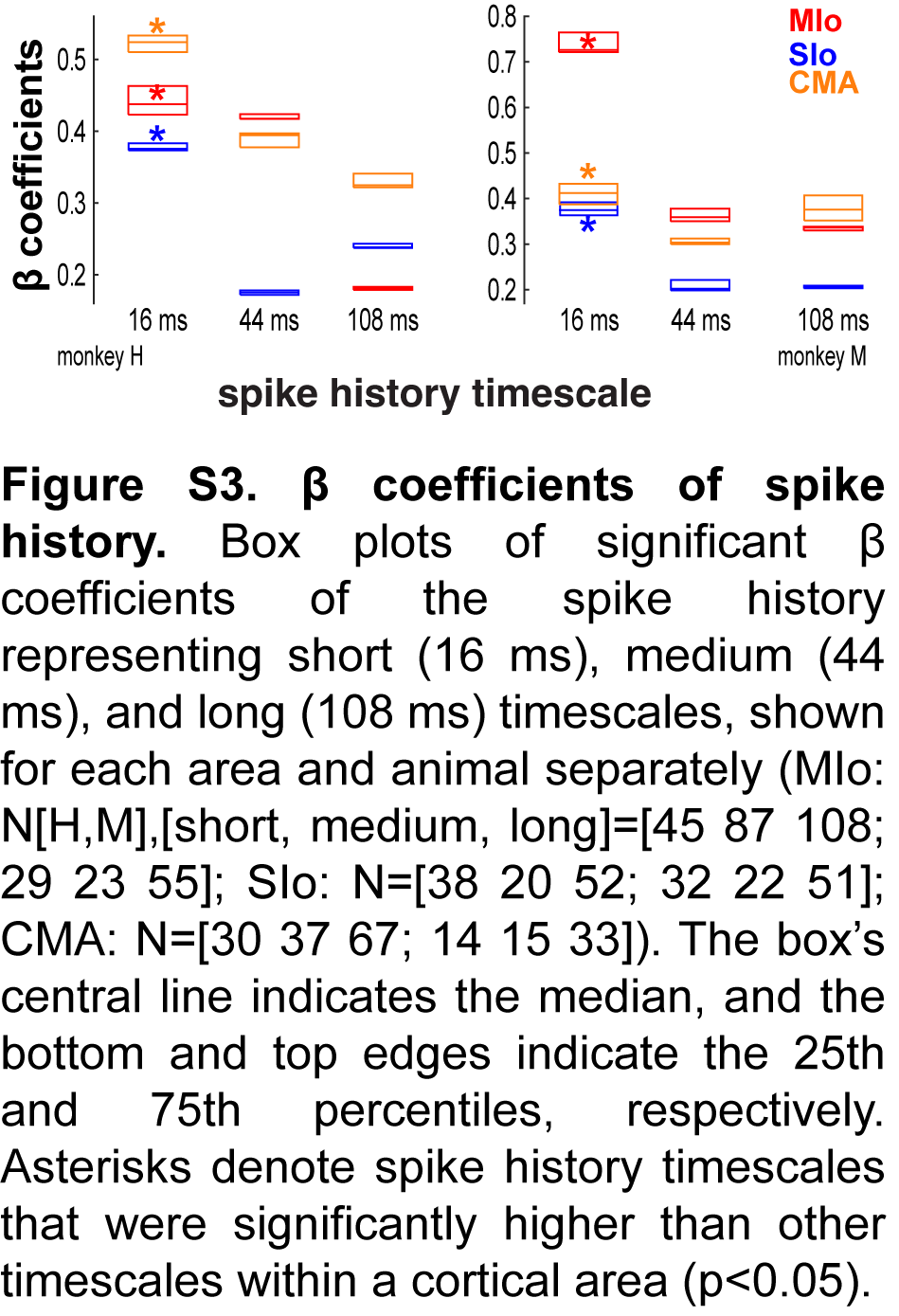

Supplement: Supplementary file 4 [file Image_3.tif]

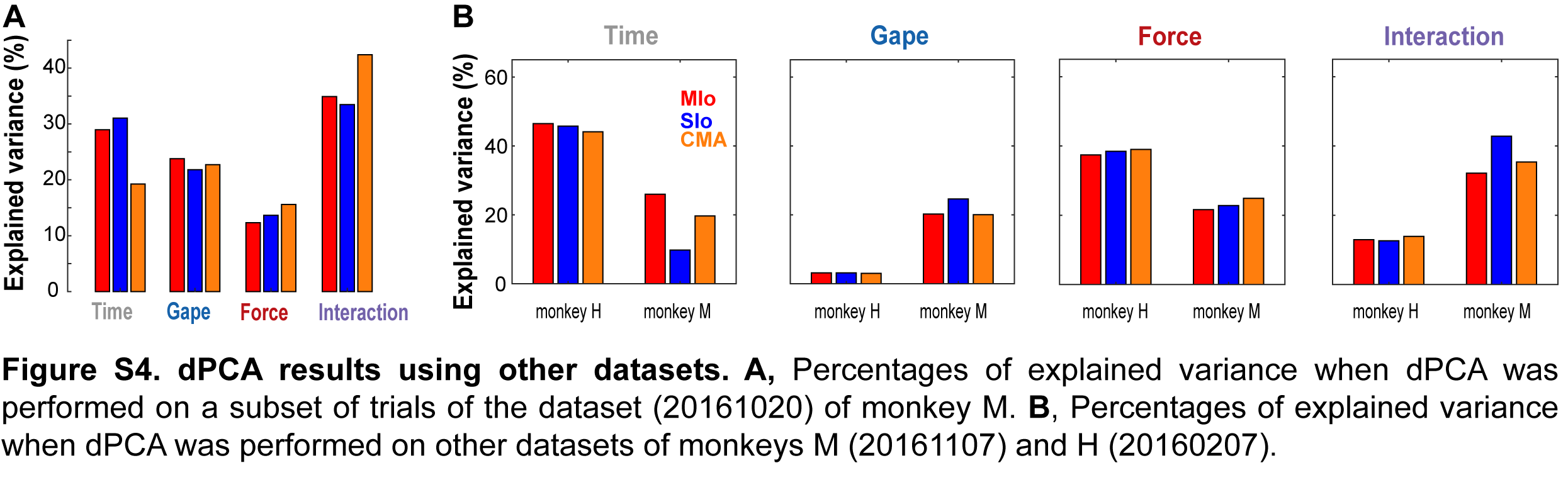

Supplement: Supplementary file 5 [file Image_4.tif]
